# Nudging children towards whole wheat bread: a field experiment on the influence of fun bread roll shape on breakfast consumption

**DOI:** 10.1186/1471-2458-14-906

**Published:** 2014-09-02

**Authors:** Ellen van Kleef, Milou Vrijhof, Ilse A Polet, Monique H Vingerhoeds, René A de Wijk

**Affiliations:** Marketing and Consumer Behaviour Group, Wageningen University, Hollandseweg 1, Wageningen, The Netherlands; Wageningen UR-Food and Biobased Research, Consumer Science and Intelligent Systems, Bornse Weilanden 9, Wageningen, The Netherlands

**Keywords:** Nudge, Nudging, Whole grain, Whole-wheat bread, Children’s food preferences, Bread shape, Visual appeal

## Abstract

**Background:**

Many children do not eat enough whole grains, which may have negative health consequences. Intervention research is increasingly focusing on nudging as a way to influence food choices by affecting unconscious behavioural processes. The aim of this field study was to examine whether the shape of bread rolls is able to shift children’s bread choices from white to whole wheat during breakfast to increase whole grain intake.

**Methods:**

In a between-subjects experiment conducted at twelve primary schools in the Netherlands, with school as the unit of condition assignment, children were exposed to an assortment of white and whole wheat bread rolls, both varying in shape (regular versus fun). Children were free to choose the type and number of bread rolls and toppings to eat during breakfast. Consumption of bread rolls was measured at class level via the number of bread rolls before and after breakfast. In addition, children (N = 1113) responded to a survey including questions about the breakfast.

**Results:**

Results of the field experiment showed that about 76% of bread consumption consisted of white bread rolls. Consumption of white bread rolls did not differ according to shape (all P-values > 0.18). However, presenting fun-shaped whole wheat bread rolls almost doubled consumption of whole wheat bread (P = 0.001), particularly when the simultaneously presented white bread rolls had a regular shape (interaction P = 0.02). Survey results suggest that slight increases in perceived pleasure and taste are associated with these effects.

**Conclusions:**

Overall, presenting whole wheat bread in fun shapes may be helpful in increasing consumption of whole wheat bread in children. Future research could examine how improving the visual appeal of healthy foods may lead to sustained behaviour changes.

## Background

Whole grains have various health benefits [1–4]. For example, children who consume whole grains have significantly higher intakes of fibre and magnesium compared to children who do not consume whole grains [5]. Hence, international nutrition guidelines advise to replace refined grains with whole grains. Unfortunately, in many countries intake of whole grains by children and adults is far below the recommended level [6–9]. Clearly, even though the health benefits of sufficient whole grain consumption are substantial, people do not always make food choices that serve their best (long-term) interests. Some of the reasons for insufficient consumption of whole grains are the less preferred taste, higher price (in some situations) and lack of availability [10]. Appearance and taste of whole grain foods such as bread have been shown to be particularly important choice criteria for consumers [11]. Furthermore, misunderstanding about sources of whole grain exists in that some consumers believe that the darker the bread, the healthier it is, although this is not necessarily true as some breads are colored using dark caramel, molasses or other products.

Various efforts have been made to increase the consumption of whole grain foods. It is often assumed that education is effective in improving knowledge and that this improved knowledge will change eating behaviour of consumers. However, in general this approach has had limited success [12], because people tend to be influenced by various biases and cues in their environment when making decisions. Hence, education alone may not lead to significant sustained behavioural changes.

Building on insights from behavioural economics and psychology, nudges are environmental interventions that adapt the environment in which people make choices to help them make better choices, without appealing to people’s reason or forcing certain choices upon them [13]. Subtle characteristics of the environment influence the choices that people make, often without their awareness. Examples of nudges to increase the likelihood that consumers select healthier options include changes of the position of foods on menus [14], snack assortment presentation near the checkout [15] and food order in buffet lines [16]. Nudges to increase children’s intake of whole wheat bread could be inspired by current food marketing strategies aimed at children. Marketing studies have shown that children usually respond positively to foods and beverages that are specifically targeted at them, so-called ‘fun food’. Fun foods are developed to appeal to children because of their shape, package, cartoon images, merchandise tie-ins, or colours and language referring to fun [17]. However, the majority of foods specifically targeted at children often have a poor nutritional composition, such as being high in sugar, fat and energy [17, 18].

The question remains how proven marketing techniques to promote unhealthy products can be ‘borrowed’ from the food industry to change eating habits in a more healthy direction. In general, the visual appearance and shape of food is a very important factor in eating behaviour [19]. Research on hedonic consumption has shown that consumers experience greater pleasure when products have higher aesthetic qualities. Moreover, consumers’ expectations of a product and their engagement with the product can influence enjoyment both before and during consumption [20]. Shape may provide more fun, pleasure or excitement in the choice. However, the few studies that looked at shape as a way to encourage consumption of healthy foods among children show mixed results. One study showed no effect of the shape of high-fibre snacks (banana bread, pancakes and sandwiches) on consumption [21]. These snacks were served either in normal (round, square) or shaped (heart, hands, animals) form to pre-schoolers (aged 2–5 years) during a 9-week period. Two other studies showed that an appealing shape is able to increase consumption of fruit and vegetables. Jansen and colleagues [22] studied the effect of the visual appeal of fruits on their consumption by 4–7 year old children. Piercing fruits with flagged cocktail sticks and sticking them into a watermelon created visually appealing fruits. Regular fruits were offered on a white plate. Results showed that children ate about twice as much of the visually appealing fruit mix as opposed to the regular fruit mix. Olsen and colleagues [23] served children raw snack vegetables in various shapes and serving styles. Shape appeared to have a large effect on intake. Likewise, based on a series of focus groups with children, Elliott [24] showed that fun elements are very appealing to children. Younger children are particularly attracted by tie-in merchandizing and fun shapes, while older children consider the overall look of the food packaging.

No work has been done on the effect of shape of bread on bread selection and intake by children. Can changing the shape of bread into a fun shape have an effect on what and how much children eat? Does shape work just as well in changing choices in whole wheat bread and white bread? Hence, the aim of this study is to examine whether the shape of bread rolls can stimulate consumption of whole wheat bread when children have the choice between whole wheat bread rolls and white bread rolls. The experiment was conducted in twelve primary schools during a festive breakfast event.

An important characteristic of nudging is that people continue to have freedom of choice. Therefore, in our study children had the freedom to choose white bread. Both white and whole wheat bread rolls were presented and varied in shape based on a between-groups design. This design allowed examining the relative impact of shape in both white and whole wheat bread in influencing choices. Whole grain bread consumption in children was expected to increase when the bread rolls were presented in a shape that might be visually attractive to children compared to presentation in a regular shape. We also expected an interaction between the shape of white bread and the shape of whole wheat bread. That is, we hypothesized that the largest increase in whole wheat bread consumption would occur in the condition where the fun-shaped whole wheat bread rolls are combined with regular shaped white bread rolls.

## Methods

In this study, the primary data collected was the bread roll consumption per class during the field experiment. The field experiment is described below. In addition, we also collected survey data from the children. This part of the study is described in a separate section (see Survey).

### Field experiment

The experiment was conducted in the first week of November 2013 (Tuesday 5^th^ November to Friday 8^th^ November) during the National School Breakfast event. The National School Breakfast is a yearly event in the Netherlands in which about 400,000 children from about 2000 primary schools have breakfast at school. The National School Breakfast event was organized by the Dutch Bakery Center (Nederlands Bakkerij Centrum), a foundation that advices the Dutch bakery industry and provides up to date knowledge to bakers as well as health professionals and consumers. The aim of the National School Breakfast event is to promote healthy breakfast habits among children. Various Dutch food companies, retailers and bakeries supply the breakfast foods and drinks directly to the schools, free of charge. The Dutch Nutrition Centre is an official supporter of the event. Schools can sign in at a special website and join the event for €0.25 per participating child. This money is donated to charity. The University Social Science Ethical Review board of Wageningen University approved this study.

### Schools and classes

Between four and twelve weeks before the event, 33 randomly selected primary schools in the center region of the Netherlands (provinces Utrecht and Gelderland) were called to ask whether they were willing to participate in the study with all children in grades five to eight. These schools were selected from a list of schools that had registered to join the National School Breakfast event. Staff at schools had to be willing to have children eat the breakfast in their own class room and not, for example, combine classes and eat together in the school hall. Fourteen schools agreed to participate in the study. Two schools were declined participation as they did not have enough classes that could take part. Each of the twelve schools participated with three to five classes, making the total number of classes that participated 47 (Table 1). Children in these classes were eight to twelve years old and attended these schools. This particular age group was selected as children are increasingly able to choose their own type of bread and are able to fill in a simple questionnaire. Teachers and parents were informed about the project through a letter. Parents could refuse participation of their child, but none of the parents did.

**Table 1:** **Random assignment of schools and classes to four conditions**

***Condition***	Number of participating schools	Number of classes	Number of classes with younger children (combination grade 4/5 till grade 6*)	Number of classes with older children (combination grade 6/7 till grade 8*)
Regular shaped whole wheat bread and regular shaped white bread	3	12	5	7
Regular shaped whole wheat bread and fun-shaped white bread	3	12	5	7
Fun-shaped whole wheat bread and regular shaped white bread	3	11	5	6
Fun-shaped whole wheat bread and fun-shaped white bread	3	12	6	6

*In the Netherlands, primary schools have 8 grades starting at grade 1 with 4-year-old children.

### Design

Schools, participating with multiple classes, were randomly assigned to one condition of a two (shape of white bread rolls provided: regular or fun) by two (shape of whole wheat bread rolls provided: regular or fun) between-subjects experimental design. All classes within a school were assigned to the same condition to prevent mistakes and awareness of the shape manipulations. In all classes, the children were provided with a more than sufficient supply of white and whole wheat bread rolls.

### Procedure

**Figure 1:**
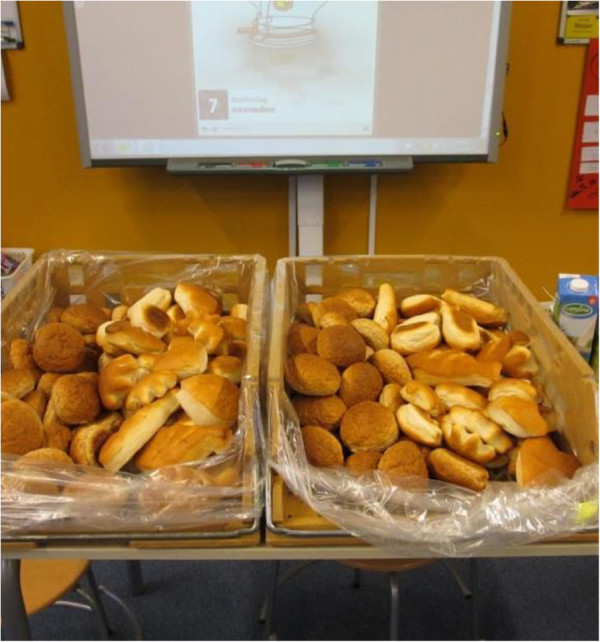
**Presentation of bread rolls in classroom.**

In the weeks before the breakfast session, schools received an information leaflet explaining the goal and procedures of the study. The afternoon before the breakfast, all breakfast products and deep-frozen bread rolls were delivered to the schools.Breakfast was served when the children arrived at school. The children had the breakfast with their own classmates in their own classroom. Bread rolls were presented in two baskets in front of the classroom. Each basket contained the two types of bread rolls in equal numbers: whole wheat bread and white bread in the shape according to the experimental design (see Figure 1). The position of the whole wheat bread rolls in the baskets (left or right) was randomly varied across classes. At the start of the breakfast children were instructed to select one bread roll, and were told that they could later return for more as many times as they wanted. They could also freely decide what type of topping or spread to put on the bread rolls and what to drink. The children were instructed to leave unfinished bread rolls on their plate and not to throw any leftovers in the trash. The teachers were instructed to refrain from influencing the eating behaviour of the children or explaining the goal of the study. In general, breakfast took about 45 to 60 minutes and the teachers were free to decide the way in which the children were seated, and the type of dishware used. For example, in some classes, children sat in small groups and in other classes, there was one larger table for all children in that class. It was not allowed, however, to eat together with other classes or to exchange bread rolls with them. After breakfast, children were asked to fill in a questionnaire (see Survey).

### Bread rolls and additional product supply

**Figure 2:**
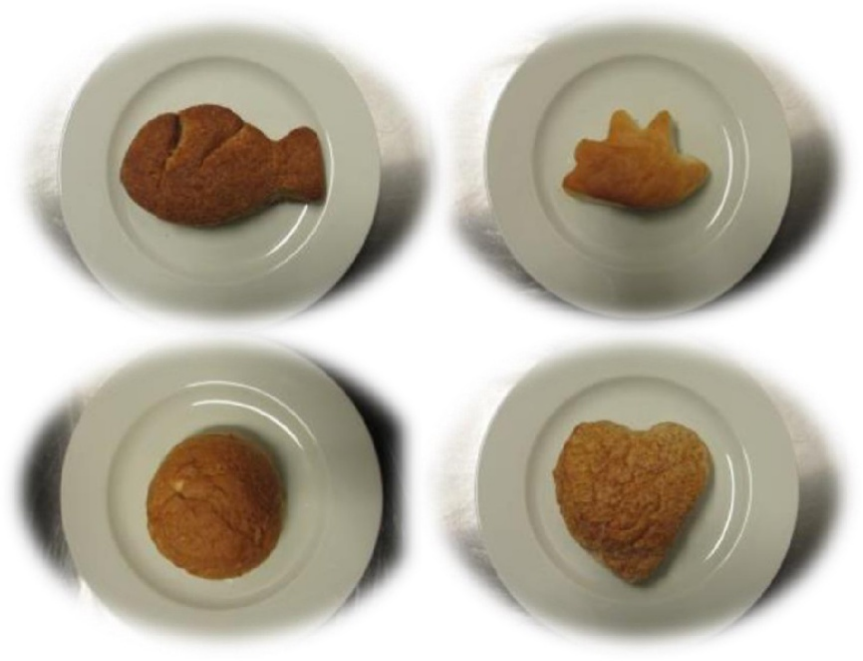
**The four types of bread roll shapes included in the study.** Left below is the regular round shape.

Three fun bread roll shapes were developed based on discussion among the researchers and the bakers: heart shape, fish shape and hand shape (Figure 2). The bread rolls were baked by the Dutch Bakery Centre with standardized recipes. White bread rolls had ingredients in the following proportions: 100% white flour, 1.5% salt, 15% bread improver (mix of additives to enhance the bakery process), 6% yeast and 60% water. Whole wheat bread rolls were prepared with the same recipe using whole wheat flour and a bit more water (65% of the total amount of flour). The whole wheat flour contained partly coarse and partly fine brans.

We made sure that no shortage of the different bread rolls would occur. Each class with 25 children or less got 100 white bread rolls and 100 whole wheat bread rolls to ensure that about four bread rolls of each type were available per child. Where more than 25 children were present in a class, additional bread rolls were available to refill when needed.

In each class, researchers also weighed three randomly selected bread rolls from each type of bread (whole wheat and white) and shape (regular, hand, heart and fish shape) to calculate the average weight of each type of bread roll. The average weight of a whole wheat bread roll was 47.2 grams (SD = 4.2) for regularly shaped and 37.6 grams for fun-shaped (SD = 2.3). Fun-shaped white bread rolls were the heaviest on average. Differences in weight between the shapes existed. The average weight of a white bread roll was 45.3 grams (SD = 1.4) for regularly shaped and 54.6 grams for fun-shaped (SD = 4.0).

In addition to the supplied bread, each class was provided with a standard set of breakfast foods and drinks. Each group of 25 children received one tub of margarine, one jar of apple-cinnamon syrup, four pots of cheese spread, one packet of chocolate sprinkles, one jar of strawberry jam, one packet of 30+ Gouda cheese, three litres of semi-skimmed sterilized milk and two litres of orange juice. Teachers were asked to use only these breakfast products although a few teachers also offered other breakfast foods or drinks, such as tea. In the classes only the bread supplied for this study was available.

### Measures

The bread rolls available for each class were counted and the number was recorded together with the total weight of randomly selected white and whole wheat bread rolls. After the breakfast, leftovers were collected and researchers noted the number and type of leftover bread rolls (whole wheat or white) for each class. The number of bread rolls consumed per class was calculated by subtracting the number of leftover bread rolls from the total number of bread rolls available of each type (whole wheat and white). For each class, researchers recorded the number of children that were present and the number of children that actually took part in the breakfast. For example, one child did not eat any of the offered breakfast due to allergies and a few other children ate nothing because of other health problems (e.g. feeling sick).

### Data analysis

We calculated the number of consumed bread rolls per class by subtracting the number of leftover bread rolls (entire rolls and leftovers at plates) from the total number of bread rolls available before breakfast. Leftover breads were not weighed but assessed as entire, half, three-quarter or one quarter leftovers. We also subtracted the number and type of bread rolls (whole wheat or white) eaten by the teachers. The average consumed number of both white and whole wheat bread rolls per child was calculated by dividing the total number of bread rolls consumed by the class by the total number of children that participated in the breakfast. In one class belonging to the condition with both fun white and whole wheat bread rolls, it was not possible to count the number of leftover bread rolls as they were accidentally mixed up with other (already counted) leftover bread rolls. Therefore, data from this class was removed from the analysis of the field experimental data, leaving data from 46 classes in the data set.

Statistical analyses of the consumption data were performed using analyses of variance (ANOVAs) with the average number of whole-wheat bread rolls eaten by a child in a particular class as the dependent variable, and shape of whole-wheat offered (two levels: fun versus regular) and shape of white bread offered (two levels: fun versus regular) as independent variables. Separate ANOVAs were run with total number of bread rolls consumed and average number of white bread rolls consumed as the dependent variable. P-values smaller than 0.05 were considered to indicate statistical significance. Effect sizes (η_p_^2^) are reported for statistically significant outcome measures and can be interpreted as an estimate of the proportion of variance attributable to the factor considered. An effect size of 0.2 indicates a small effect, 0.5 indicates a moderate effect, and an effect size of 0.8 is considered large.

### Survey

Immediately after finishing the breakfast, children were asked to give their opinion about the breakfast and to write down the number and type of bread rolls consumed during the breakfast. These self-reported bread consumption data were used to compare observed and self-reported data.

### Design, procedures and participants

All participating children were asked to fill in a questionnaire about their breakfast experience. Participants were 1113 children from 47 classes in twelve schools. The survey was completed after the breakfast. Instructions on top of the questionnaire stated that there were no right or wrong answers. Children took approximately ten minutes to complete the questionnaire. For the youngest children, several teachers explained how to fill in the questionnaire.

### Measures

The taste experience was measured using two items: ‘The breakfast was tasty’ and ‘The bread rolls were tasty’. ‘Before I went to school I already had breakfast at home’ and ‘I had a strong appetite before we started eating’ were included to control for feelings of hunger and appetite. ‘It was nice to choose a bread roll’ was included to capture the pleasure in making a bread roll choice. We also checked whether children noticed our manipulations by including the item ‘The bread rolls looked funny’. We included ‘I ate more breakfast at school than usually at home’ to measure potential effects on perceived consumption. Each statement was scored on a scale from one (not at all) to five (very much). The statements were preceded by the phrase ‘Show your opinion by crossing the correct box’. To make the response format more child friendly, smiley faces were added to the answer categories.

Children were asked to indicate the frequency with which they have breakfast at home on an eight-point answer format ranging from ‘never’ to ‘every day’. Children were also asked to indicate what they drank during the breakfast. Response categories for this question were ‘I did not drink anything’ , ‘milk’ , ‘juice’ , ‘water’ and ‘other, which is….’ with space to write down what they drank.

The next question asked ‘Which bread rolls did you chose for breakfast here at school? Fill in the numbers’. Depending on the condition, children could write down the number of bread rolls of each available specific shape. Shapes that were not available in that condition were not mentioned. They could also answer ‘do not know’. Children also reported age and gender. Finally, children had the opportunity to write down anything that they wanted about the breakfast or the questionnaire in an empty box at the end of the questionnaire.

### Data analysis

Using ANOVAs we checked whether there were differences between conditions in age, gender and appetite (before breakfast) of the children and whether they had breakfast at home before coming to school. The primary ANCOVA examined the effect of our independent variables ‘shape of whole wheat bread’ and ‘shape of white bread’ on children’s responses to the survey measures. Self-reported numbers of bread rolls consumed as indicated by the children were also examined using ANCOVAs. Age and responses to the item ‘Before I came to school I already had breakfast at home’ were added as covariates to all analyses to control for their influence (see Randomisation check in results section). As large sample sizes increase the chance that very small differences between groups become statistically significant, we also report effect sizes for relevant outcome measures.

## Results

### Results field experiment

#### Total number of bread rolls consumed

On average, children ate 2.9 (SD = 0.8) bread rolls in total, which was not influenced by the manipulations in shape and type. This was revealed by an ANOVA with the total number of bread rolls consumed by each child as the dependent variable and shape of white bread rolls and shape of whole wheat bread rolls as the independent variables. Results showed no main effect of shape of white bread (F(1,42) = 3.7, P = 0.06), no main effect of shape of whole wheat bread (F(1,42) = 1.7, P = 0.20) and no interaction effect between the two variables (F(1,42) = 0.23, P = 0.64).

#### Number of white bread rolls consumed

The majority of the bread rolls consumed consisted of white bread rolls. On average, each child consumed 2.2 (SD = 0.5) white bread rolls, which equals about 76% of the consumed bread. The shape of the bread did not affect the average number of white bread rolls consumed by each child (main effect shape of white bread F(1,42) = 1.7, P = 0.19; main effect shape of whole wheat bread F(1,42) = 1.3, P = 0.26; interaction effect F(1,42) = 1.8, P = 0.18).

#### Number of whole wheat bread rolls consumed

**Figure 3:**
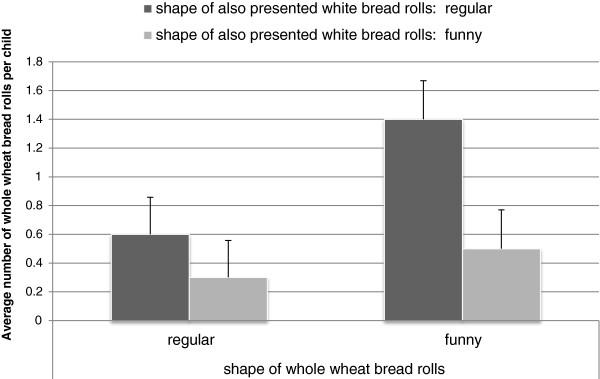
**Average number of whole-wheat bread rolls consumed by children depended on the shape of provided white and whole wheat bread rolls (based on observed bread rolls consumption data per class).**

The consumption of whole wheat bread rolls differed across the conditions (Figure 3). An ANOVA with average number of whole wheat bread rolls consumed by each child as the dependent variable and shape of white bread rolls and shape of whole wheat bread rolls as independent variables revealed a main effect of shape of white bread on the average number of whole wheat bread rolls consumed (F(1,42) = 22.0, P < 0.001; η_p_^2^ = 0.34). This means that consumption of whole wheat bread was higher when the shape of white bread rolls was regular (Mean = 1.0, SD = 0.7) compared to fun (Mean = 0.4, SD = 0.3). There was also a main effect of shape of whole wheat bread rolls (F(1,42) = 12.0, P = 0.001; η_p_^2^ = 0.22) on the average number of whole wheat bread rolls eaten. The number of whole wheat bread rolls consumed almost doubled from on average 0.5 bread rolls (SD = 0.4) when the rolls had a regular shape, to on average 0.9 bread rolls (SD = 0.7) when the rolls had a fun shape. In addition, an interaction between shape of white bread and shape of whole wheat bread was observed (F(1,42) = 5.5, P = 0.02; η_p_^2^ = 0.12). Consumption of whole wheat bread rolls was highest in the condition in which these bread rolls had a fun shape and there was no competition from fun-shaped white breads (see also Table 2 and Figure 3).

**Table 2:** **Observed and self-reported number of bread rolls consumed per child and children’s breakfast appraisal in survey (mean, SD)**

	Regular shaped whole wheat bread		Fun-shaped whole wheat bread		P-value main effect shape of whole wheat bread	P-value main effect shape of white bread	P-value interaction effect
	Regular shaped white bread	Fun-shaped white bread	Regular shaped white bread	Fun-shaped white bread			
*Observed consumption (per child)*							
Total number of bread rolls	3.0 (0.7)	2.6 (0.8)	3.3 (0.9)	2.8 (0.4)	0.20	0.06	0.64
Number of whole wheat bread rolls	0.6 (0.5)	0.3 (0.3)	1.4 (0.6)	0.5 (0.2)	0.001	<0.001	0.02
Number of white bread rolls	2.3 (0.4)	2.3 (0.6)	2.0 (0.7)	2.4 (0.3)	0.26	0.19	0.18
Children’s breakfast appraisal*							
I ate more breakfast at school than usually at home	3.7 (1.5)	3.6 (1.5)	3.7 (1.5)	3.6 (1.5)	0.89	0.44	0.84
The bread rolls looked funny	2.2 (1.4)	4.3 (1.0)	4.1 (1.3)	4.5 (0.9)	<0.001	<0.001	<0.001
It was enjoyable to choose a bread roll	3.4 (1.3)	3.7 (1.3)	3.6 (1.2)	4.1 (1.0)	<0.001	<0.001	0.76
The breakfast was tasty	4.6 (0.7)	4.3 (1.1)	4.5 (0.9)	4.7 (0.7)	0.04	0.46	<0.001
The bread rolls were tasty	4.6 (0.8)	4.4 (1.0)	4.6 (0.8)	4.5 (0.8)	0.29	0.01	0.14
*Children’s self-reported consumption*							
Total number of bread rolls	3.6 (2.0)	3.3 (2.5)	3.8 (2.6)	3.3 (1.9)	0.81	0.045	0.65
Number of whole wheat bread rolls	1.0 (1.7)	0.4 (0.9)	1.5 (2.0)	0.7 (1.4)	<0.001	<0.001	0.28
Number of white bread rolls	2.3 (2.0)	2.9 (2.4)	2.0 (2.1)	2.4 (1.7)	<0.01	<0.001	0.53

*Note: responses are measured on five-points scales, ranging from 1 (not at all) to 5 (very much).

#### Consumption analyses based on estimated average grams of bread consumed

To account for differences in the weight of bread rolls of different types, similar analyses were carried out with the estimated average grams of whole wheat bread consumed per child as dependent variables, based on the class level average weights of the available bread rolls. For one class, these data were not available, so this leaves 45 classes in the dataset. The pattern of results of the ANOVA with estimated grams of whole wheat bread consumed per child as the dependent variable and shape of whole wheat bread and shape of white bread as independent variables was similar to the ANOVA with average number of whole wheat bread rolls consumed. There was both a main effect of shape of white bread rolls (F(1,41) = 17.9, P < 0.001; η_p_^2^ = 0.30) and a main effect of shape of whole wheat bread rolls (F(1,41) = 9.4, P < 0.01; η_p_^2^ = 0.19). For example, children ate on average 19 grams of whole wheat bread rolls when their shape was regular compared to 35 grams when their shape was fun. The interaction between the two factors was also significant (F(1,41) = 6.0, P = 0.02; η_p_^2^ = 0.13). Most whole wheat bread (51 grams) was consumed in the condition in which children could choose between fun-shaped whole wheat bread and regularly shaped white bread rolls.

### Results survey

1113 children filled in the questionnaire (555 girls, 518 boys, and gender not filled in by 40 children). Their age ranged from six to thirteen with a mean of 9.7 years (SD = 1.2). On average, children reported to have breakfast at home 6.6 times per week (SD = 1.3). During the school breakfast, 97% of the children indicated that they drank something. Almost 51% of the children reported juice as a drink and 42% milk.

#### Randomisation check

Because schools had been randomly assigned to conditions, we first checked whether gender, age, appetite and earlier breakfast varied across conditions. There were no significant differences between the conditions for gender (χ = 0.20, P = 0.35) and appetite before eating (all P values > 0.12). However, significant differences across conditions were observed for age (interaction between shape of wheat bread and shape of white bread, F(1,1084), 9.8, P < 0.01) and the item ‘Before I came to school I already had breakfast at home’ (main effect shape of white bread F(1,1088) = 21.7, P < 0.001; interaction between shape of whole wheat bread and white bread F(1,1088) = 8.1, P < 0.01). Therefore, we control for the influence of these two variables in the analysis of the self-reported survey data by including them in all statistical analyses as covariates.

#### Influence of bread roll shape on enjoyment and perceived tastiness

There was no difference across conditions in children’s response to the item ‘I ate more breakfast at school than usually at home’ (all P values > 0.44). The shape of the bread rolls in the different conditions, however, did not go unnoticed. The bread rolls were considered to be fun when one of the two options was of fun shape and were perceived as most fun when both options had a fun shape (all P values < 0.001, effect size shape of white bread η_p_^2^ = 0.23; effect size shape of whole wheat bread η_p_^2^ = 0.16, and effect size interaction effect η_p_^2^ = 0.13; see Table 2). A fun shape of either white or whole wheat bread rolls also made it more enjoyable to choose a bread roll (main effect white bread shape P < 0.001; η_p_^2^ = 0.03; main effect whole wheat roll shape P < 0.001; η_p_^2^ = 0.02; no interaction effect, P = 0.76).

Our shape manipulations also impacted the perceived tastiness of the bread rolls and the entire breakfast. Breakfast tasted slightly better when the whole wheat bread rolls had a fun shape (M = 4.6, SD = 0.9) compared to when the shape was regular (M = 4,5, SD = 0.9; P = 0.04; η_p_^2^ = 0.004). The shape of the white bread rolls had no influence (P = 0.46). There was also an interaction effect between the two shape manipulations (P < 0.001; η_p_^2^ = 0.02) showing that the breakfast was slightly more tasty when both bread rolls had a fun shape. The bread rolls themselves were equally tasty across conditions, except that when white bread rolls were regularly shaped there was a higher score on taste (M = 4.6, SD = 0.8) than when these white bread rolls were fun-shaped (M = 4.4, SD = 0.9; P = 0.01).

#### Children’s self-reported number of bread rolls consumed

Of all children’s questionnaires, in 209 of them (18.8%) data about the number of consumed bread rolls were missing as children only checked the box to indicate the type of bread they consumed instead of noting the number of bread rolls consumed. For a number of children we only have data about one type of bread and the data about the other type of bread is missing. Children were not asked to report the total number of bread rolls consumed. Consequently, adding the self-reported number whole wheat bread rolls to the self-reported number of white bread rolls does not equal the total number of bread rolls consumed (see Table 2).

On average, children reported to have consumed a total of 3.5 bread rolls (SD = 2.3). This number is more than half a bread roll higher than what was estimated based on observed class level data. An ANCOVA with these self-reported total number of bread rolls consumed as the dependent variable and shape of white bread rolls and shape of whole wheat bread rolls as independent variables revealed a main effect of shape of white bread (F(1,862) = 4.0, P = 0.045; η_p_^2^ = 0.01). Children reported to have consumed on average more bread rolls in total when the white bread rolls had a regular shape (M = 3.7, SD = 2.3) than when the white bread was fun-shaped (M = 3.3, SD = 2.2). No main effect of shape of whole wheat bread (F(1, 862) = 0.1, P = 0.81) or interaction between the two independent variables (F(1,862) = 0.2, P = 0.65) was observed.

On average, children reported to have consumed 2.4 white bread rolls (SD = 2.1). In contrast to the results based on observed consumption data, the shape of the bread rolls did impact the average number of white bread rolls consumed by children (main effect shape of white bread F(1,886) = 13.9, P < 0.001, η_p_^2^ = 0.02; main effect shape of whole wheat bread F(1,886) = 8.5, P < 0.01, η_p_^2^ = 0.01; interaction effect F(1,886) = 0.4, P = 0.53). White bread rolls were most popular when the whole wheat bread rolls were regularly shaped and when white bread rolls were funny-shaped.

On average, children reported consumption of 0.9 whole wheat bread rolls (SD = 1.6). Similar to the analyses based on observed bread roll consumption, the shape of bread rolls influenced the type of bread roll selected. An ANCOVA with the self-reported number of eaten whole wheat bread rolls as the dependent variable and shape of white bread rolls and shape of whole wheat bread rolls as independent variables revealed both a main effect of shape of white bread (F(1,972) = 51.3, P < 0.001, η_p_^2^ = 0.05) and whole wheat bread (F(1,972) = 21.3, P < 0.001, η_p_^2^ = 0.02). The number of whole wheat bread rolls eaten increased from 0.6 when whole wheat bread rolls had a regular shape (SD = 1.4) to 1.1 when the whole wheat bread was fun-shaped (SD = 1.8). Moreover, children reported substantially less consumption of whole wheat bread when the white bread rolls had a fun shape (M = 0.5, SD = 1.2) compared to when the white bread rolls had a regular shape (M = 1.2, SD = 1.9). No interaction between the two independent variables (F(1,972) = 1.2, P = 0.28) was observed. The covariate age (F(1,972) = 4.1, P = 0.04) had a significant impact on the number of whole wheat bread rolls consumed. The covariate ‘before I came to school I already had breakfast’ (F(1,972) = 0.1, P = 0.74) had no main effect on the number of whole wheat bread rolls consumed. The pattern of significance of the two independent variables did not change when running the analysis without the two covariates.

## Discussion and conclusion

The aim of this study was to investigate whether children’s consumption of whole wheat bread increases when it is offered in a fun shape. This study demonstrated that the shape of bread rolls significantly influenced the bread choices children make. Consistent with our expectations, whole wheat bread roll consumption increased when it had a fun shape. In particular, consumption of whole wheat bread rolls was the highest when they had a fun shape while the presented ‘competing’ white bread rolls had a regular shape. In other words, when children have the choice between a fun-shaped whole wheat bread roll and a regular shaped white bread roll, the popularity of the first option increases. Although the magnitudes of the effects were small, fun shapes of bread rolls enhanced children’s liking of the entire breakfast and led to slightly more pleasure in choosing. The shape may have attracted children’s attention and children may enjoy trying out unfamiliar shapes of bread rolls.

A key strength of this study is the experimental design. In contrast to most consumption studies in which there is no choice between different types of food within a condition, in our study children could always choose between white and whole wheat bread. This not only allowed us to show consumption effects due to type of bread, but it also provided insight into whether our shape manipulation was strong enough to change the trade-off between white and whole wheat bread.

We measured bread consumption both objectively by counting the number of bread rolls at class level and subjectively by using children’s self-reported numbers of bread rolls in the survey. Overall, the pattern of results was quite similar in that children responded to the shape manipulations. However, self-reported total bread consumption by children was higher than actual consumption observations in the classroom. Research on children’s recall of food intake has shown that considerable misreporting is likely. The cognitive ability to self-report food intake has often not yet been developed, leading to both underreporting and overreporting [25]. Children may forget what they ate or give socially desirable answers [26]. In the current school setting, peer pressure cannot be ruled out.

### Limitations

We used the school as unit of assigning conditions and the class as unit of measurement and analysis. In this way, children could freely choose and eat bread rolls without being disturbed by researchers monitoring each individual child’s consumption. A drawback of this approach, however, is that confounding factors may have influenced the outcomes. It may be that schools differed in ways that influenced results. All schools were located in the centre region of the Netherlands, but school differences in socio-economic status may be present. These differences may have confounded the results, since we did not measure and take into account this and other variables at an individual or school level.

The experiment took place in a natural setting, although school is not a typical setting for children to have breakfast. The National School Breakfast is usually experienced as a very festive event. As such, it is not similar to a common breakfast in a family context at home where parents often serve breakfast. This festive atmosphere may explain the popularity of white bread rolls.

Limitations of this experiment are that the consumption of extra foods and drinks included in the breakfast was not measured and that almost 20% of children’s self-reported data on how many bread rolls were eaten was missing. This may have influenced the results. Moreover, the weight of some types of bread rolls differed. Although the pattern of results of whole wheat bread consumption is the same when looking at estimated weight, this might have influenced children’s choices. However, it has been shown that people base portion size decision on units rather than weights as one unit represents a normal consumption amount [27]. When this mechanism occurs, weight may have been of less importance.

### Implications

It is important to mention that although small nudges such as changing the shape of bread rolls can shift children’s choices, this study shows that when having the choice between whole wheat and white bread, white bread remains the most popular option for children. The great popularity of white bread suggests that we need to better understand whether these preferences for white bread are related to the sensory characteristics of bread (e.g. colour, smell, taste) or to other variables, such as limited availability in normal breakfast situations.

Producing fun foods or marketing foods specifically at children has been criticized as irresponsible in light of the current childhood obesity problems worldwide [24, 28]. The argument is that children should not base their food preferences on entertainment, but should also learn to eat ‘adult’ foods. For unhealthy foods, fun marketing is a common and effective promotion technique. However, the same underlying principle of fun can also work for the promotion of healthier foods. Recent research suggests that this may be a better strategy than promoting foods to children based on their health value. Health messages related to food may lead to inferences that the food is not very tasty and as a result, children may consume less of it [29].

Changes in consumption such as the ones found in this study may be short-lived. The fun derived from the novelty of the bread rolls may wear off over time. However, using proven marketing techniques to encourage healthy food consumption may have the advantage of giving children the necessary nudge to try something new. An idea might be to introduce a new whole wheat shape on a regular basis (‘fun shape of the month’) to keep children’s engagement high. It has been shown for other foods such as vegetables that offering food repeatedly is sufficient to increase intake over time as this makes new and less preferred foods slowly more familiar and liked [30]. Food preferences are partly determined by genetics, but also learned at a young age through frequent exposure. Future research could explore repeated exposure so that eating sufficient amounts of whole wheat bread becomes a habit.

It is of crucial importance to study how children’s food choice might be impacted by small contextual cues. As such, the results of this study have implications for policies and practices within school meal programs. Adapting the school food environment is increasingly seen as fundamental to help children develop healthy eating habits. Instead of requiring children to eat certain foods, improving the attractiveness of healthier options is a promising route. This study is an example of how nudging approaches may contribute to healthier food choices of children.
